# Qualitative Verification of Machine Learning-Based Burnout Predictors in Primary Care Physicians: An Exploratory Study

**DOI:** 10.1055/a-2595-0415

**Published:** 2025-09-05

**Authors:** Daniel Tawfik, Stefanie S. Sebok-Syer, Cassandra Bragdon, Cati Brown-Johnson, Marcy Winget, Mohsen Bayati, Tait Shanafelt, Jochen Profit

**Affiliations:** 1 Department of Pediatrics, Stanford University School of Medicine, Palo Alto, California, United States; 2 Department of Emergency Medicine, Stanford University School of Medicine, Palo Alto, California, United States; 3 Department of Medicine, Stanford University School of Medicine, Palo Alto, California, United States; 4 Operations, Information & Technology, Stanford Graduate School of Business, Palo Alto, California, United States

**Keywords:** physician burnout, machine learning, experiences, electronic health record usage measures

## Abstract

**Background:**

Electronic health record (EHR) usage measures may quantify physician activity at scale and predict practice settings with a high risk for physician burnout, but their relation to experiences is poorly understood.

**Objectives:**

This study aimed to explore the EHR-related experiences and well-being of primary care physicians in comparison to EHR usage measures identified as important for predicting burnout from a machine learning model.

**Methods:**

Exploratory qualitative study with semi-structured interviews of primary care physicians and clinic managers from a large academic health system and its community physician partners. We included primary care clinics with high burnout scores, low burnout scores, or large changes in burnout scores between 2020 and 2022, relative to all primary care clinics in the health system. We conducted inductive and deductive coding of interview responses using a priori themes related to the machine learning model categories of patient load, documentation burden, messaging burden, orders, and physician distress and fulfillment.

**Results:**

Interviews with 16 physicians and 4 clinic managers identified burdens related to three dominant themes: (1) messaging and documentation burdens are high and require more time than most physicians have available during standard working hours. (2) While EHR-related burdens are high they also provide patient-care benefits. (3) Turnover and insufficient staffing exacerbate time demands associated with patient load. Dimensions that are difficult to quantify, such as a perceived imbalance between job demands and individual resources, also contribute to burnout and were consistent across all themes.

**Conclusion:**

EHR-related work burden, largely quantifiable through EHR usage measures, are major source of distress among primary care physicians. Organizational recognition of this work as well as staffing and support to predict associated work burden may increase professional fulfillment and reduce burnout among primary care physicians.

## Background and Significance

Symptoms of burnout among physicians continue to rise, with recent reports suggesting that at the height of the pandemic burnout affected 68 to 76% of all primary care physicians. 1 2 This finding has sobering implications for other aspects of physician well-being, quality of patient care, access to care, and institutional costs. 3 4 5 6 7 8 9 10 11 12 13 14 15 Identifying rates of burnout traditionally has been done through survey collection, although there are increasing efforts to identify at-risk work units using prediction models that leverage ubiquitously collected electronic health record (EHR) metadata. 16 17 18 What is still missing, however, is an understanding of the relationship between these quantitative EHR measures and the experiences of physicians. Without this knowledge, it is unknown whether there are additional features that could be measured to improve the precision and accuracy of prediction models.

Routinely collected EHR metadata include a myriad of measures related to aspects of the work environment known to be associated with burnout (e.g., measures related to workload, efficiency EHR usability, and impaired work-life integration). 9 19 20 21 22 23 24 25 26 27 28 29 30 31 32 33 34 35 36 37 38 39 40 Building upon this existing research, we recently developed a burnout prediction model for primary care physicians, which identified several EHR usage measures that were limited in predicting individual burnout scores but showed promise in identifying clinics at increased risk. 16 The top ten features of this model are outlined in Table 1 .

**Table 1:** Top 10 quantitative findings from the burnout prediction model, and related key concepts and themes

Feature importance rank	Quantitative finding	Concept	Theme
1	Higher automated/administrative messages per day predict higher burnout	Automated messages	Messaging and documentation burden
2	Higher number of characters written by team members predicts higher burnout	Progress notes	Messaging and documentation burden
3	Higher provider efficiency score predicts lower burnout	Usability	EHR-related burdens
4	Higher and lower year of birth predicts lower burnout	Personal factors	Personal and professional factors
5	Higher proportion of referral orders placed using user preferences predicts lower burnout	Usability	EHR-related burdens
6	Higher minutes spent responding to medical advice requests predict higher burnout	Expanded access	EHR-related burdens
		Message triaging	Messaging and documentation burden
7	Lower number of shared smart phrases predicts lower burnout	Progress notes	Messaging and documentation burden
8	Higher proportion of other orders placed using user preferences predicts lower burnout	Usability	EHR-related burdens
9	Higher minutes spent in notes per 8 h of scheduled appointments predicts lower burnout	Progress notes	Messaging and documentation burden
		Patients vs. systems	Turnover and insufficient staffing
10	Higher proportion of characters written by copy-paste predicts higher burnout	Progress notes	Messaging and documentation burden
		Time pressure	Turnover and insufficient staffing

## Objectives

To assess the degree to which these prediction models reflect true sources of frustration for physicians, this study interviewed primary care physicians to: (1) understand the relationships between well-being and qualitative EHR-related experiences in comparison to quantitative EHR usage metrics, and (2) identify additional features relevant to well-being that may not adequately be captured in the current prediction model.

## Methods

This exploratory qualitative study used semi-structured interviews to collect data from physicians and clinic managers who work in primary care clinics within a large academic medical center or one of its community affiliates. Results are reported using the COREQ criteria. 41

### Generation of Prediction Model and Interview Guide Development

A full description of the burnout survey and prediction modeling procedures were previously published. 16 Briefly, we used institutional well-being surveys from 2019 through 2022 to identify burnout symptoms among primary care physicians, using the Emotional Exhaustion and Interpersonal Disengagement scales of the Stanford Professional Fulfillment Index, which align with the established literature for this instrument. 42 We combined these well-being survey results from 2019 and 2020 with contemporaneous EHR usage measures and employed a gradient boosting machine to generate a burnout prediction model to identify EHR usage measures that most strongly influenced the model's output.

Using this data-informed model, we developed an interview guide based on the most influential EHR usage measures, with an emphasis on identifying aspects that physicians found fulfilling and challenging about their work in the following areas: (1) patient load, (2) notes, messages, and time management, (3) orders, and (4) burnout and well-being.

### Clinic Selection and Recruitment

To inform targeted clinic recruitment using maximum variation sampling, we evaluated aggregate burnout scores by the clinic for the years 2020 and 2022. We identified the top five clinics (out of 18 candidates) in the following four categories: (1) highest average burnout scores in 2022, (2) lowest average burnout scores in 2022, (3) greatest increase in burnout scores between 2020 and 2022, and (4) greatest decrease in burnout scores between 2020 and 2022. This procedure resulted in 11 unique clinics and guided recruitment efforts. To mitigate any potential bias, study personnel involved in recruitment and interviewing were blinded to the classification of each clinic's inclusion. Physicians and managers (i.e., medical, clinic, and assistant clinic managers) were targeted to explore clinic-level factors impacting physicians' experiences. The top 6 of the 11 identified clinics were initially contacted via email. Three clinics out of the original six did not participate (two for lack of response and one for active decline), so an additional round of three clinics from clinics in high, low, and changing burnout groups were contacted.

### Data Collection

Clinic managers were asked to forward recruitment material to physicians in their clinics. Potential participants were then contacted by email up to three times by a member of the research team (CB) and asked to complete a brief in-take survey to collect demographic information and indicate preferred scheduling for interviews. Interviews were conducted via video conferencing. Participation was voluntary and participants received a $50 incentive for participation. Since this project was deemed expedited with a waiver of documentation by the Stanford IRB (#49374), each participant reviewed a consent document with a researcher prior to the interview and gave verbal consent for recording.

Interviews were in-depth and semi-structured with a topic focus on patient load (complexity and stability of panel); EHR notes, messages, and orders (burden, workflows, and time demands); physician distress (experiences of burnout and desired interventions to prevent burnout) and fulfillment; and change over time. The full interview guide is provided as Supplementary Material S1 (available in the online version only).

Interviews were conducted via Zoom (Zoom Video Communications, San Jose, California, United States), from February to June 2023 by a female member of the research team trained in qualitative research methods who had no prior relationship with the participants (CB). Interviews were audio recorded, de-identified, and transcribed verbatim by a professional transcription service (Rev, Austin, Texas, United States). Transcripts were analyzed by two members of the research team (CB-J and CB) using thematic analysis and consensus coding approaches 43 44 45 and qualitative analysis software (NVivo, QSR International, Burlington, Massachusetts, United States).

### Data Analysis

Twenty out of 74 invited participants from 6 clinics were interviewed: 16 physicians, and 4 managers (medical managers and assistant clinic managers). The nonresponse rate was 73% from the six clinics that participated.

Two of the study investigators (CB and CB-J) met three times to review the data and develop and refine the codebook, provided in Supplementary Material S1 (available in the online version only). A qualitative researcher (CB) coded deductively, identifying elements related to the verification and validation of the machine learning model, and inductively identifying themes within the data. This coding was subsequently reviewed jointly by CB and CB-J on three separate occasions and adjusted to consensus through several additional weekly discussions with the broader research team (DT, CB, CB-J, and MW) during the data analysis phase. 43 44 45 Our analytic approach also included a presentation of the findings with key informants (SSS, TS, and JP) and the institution's Physician Wellness Forum to explore whether interpretation patterns resonated with the perspectives of educational, wellness, and quality experts.

## Results

### Overview and Study Participants

We approached a total of nine primary care clinic managers representing five clinics with high or increasing burnout and four clinics with low or decreasing burnout. One clinic manager declined participation (citing a desire to protect their physicians from additional burden), two did not respond, and the participating six clinics represented two academic primary care clinics and four community affiliate clinics. Of these final six recruited clinics, three had high and increasing burnout scores, one had high stable burnout scores, one had low but increasing burnout scores, and one had low stable burnout scores. Participants consisted of 16 physicians and 4 clinic managers working in the 6 clinics and are identified using P#. Thirteen women and seven men participated.

### Major Themes

Upon achieving thematic sufficiency, we identified five themes: Messaging/Documentation Burden, EHR-related Burdens, Turnover/Insufficient Staffing, Teamwork, and Destabilizing Change. The first three of these listed themes were more developed and aligned to the study question; therefore they are described in further detail here. Of note, messaging (between patients and the healthcare team, or among members of the healthcare team) and clinical documentation represent distinct workflows with different purposes but are presented within a single theme representing EHR-mediated communication.

#### Messaging and Documentation Burden

Physicians noted that large amounts of both clinic and after-hour time were spent dealing with In Basket management (the internal messaging system within the Epic EHR). One physician participant summarized this as “the bane of a primary care doctor's experience” and noted that one of the challenges with automated messages is they are a mixture of useful and not useful information ( Table 2 ).

**Table 2:** Identified themes, related concepts, and representative quotes from interview participants, in relation to the quantitative burnout prediction model

Theme	Concept	Quote	Relation to the quantitative model	Comment
Messaging and documentation burden	Automated messages	“If one of my patients is in the emergency room, I'll get notified. If they see another physician, I'll get notified. So some of that stuff is really, really useful and I really want to know it and I want to know it immediately. But some of that stuff I don't need to know”	Concordant	Message value as determined by the physician may not correspond to message subtype as categorized by the EHR vendor
	Message triaging	“So I think medical assistants don't always have the training to be able to triage or deal with those [patient] messages appropriately. So even though we have support staff to go through the messages, the vast majority of it falls on the physician's plate”	Concordant	The value of a team-based message management approach is influenced by the training and capabilities of all involved members of the team
	Message urgency	“So the organization wanted us to send out depression screening questions […] And so that means that while I'm sitting trying to type my note at 9:00 p.m. I might get, ‘Oh, look that patient answered the questionnaire that they’re suicidal now' and now I have to deal with that without my staff. I'm not even in the office”	New insight	Message content may dictate both the urgency of response and the support resources needed by the physician to respond, independent of the message category
EHR-related burdens	Expanded access	“Yeah, the [EHR] is a pain. I think it's created a lot more access for our patients who have utilized it as another avenue for care and oftentimes for asynchronous free care. And it's created a lot of work”	Concordant	Technology-facilitated care may enable supply-demand imbalances, with healthcare demands outweighing health system resources
	Feature implementation	“[The institution] just opened this portal and literally never said, ‘Here, have some time in your day for this.’ The expectation was it's just another fire hose, deal with it. This is what patients want, or this is what we want, or I have no idea”	New insight	Response to practice changes may be influenced by physician participation in decision-making, transparent communication, and adequate support
Turnover and insufficient staffing	Patients vs. systems	“I think the last year or two, it's been a lot tougher. When I go home at 10 o'clock on Mondays, I love the patients, I don't mind working really hard for the patients. I just get so frustrated that it seems to be the system that's dragging me down”	Concordant	System inefficiencies may contribute to effort-reward imbalance
	Time pressure	“I've been so burned out. I hate going in to see patients every time. Every Sunday, I am so miserable. My kids know that Tuesday night is the best night because mommy doesn't have patient care again until Monday. That's terrible because when I'm in the visits with my patients, I like them. But it's something about the rush and all of the constraints that we have on us. So many expectations. So much of it is clerical”	Concordant	Chaotic environments and time pressure are partially attributable to administrative burden and are not fully alleviated by reduced work hours
	Turnover	“And the entire time I've been here, there has never been everybody filled and nobody leaving soon. … I don't have enough space for my patients to get in when they want … and that is, as a person who likes taking care of people, is extremely difficult”	New insight	Appointment availability may affect patient and physician satisfaction, with team instability a potential driving factor

Another participant stated, “I feel an overwhelming sense of dread as I look at the number of patient messages that I have.” Physicians tried to clear their In Basket each day, but were often unable to due to high message volume or other responsibilities, with one noting “by the time I get to my messages sometimes it's like midnight.” Many participants reported doing message management on their nonclinic days, with one stating, “I was recently on vacation for a week, and I logged in maybe three or four times to stay on top of it.”

Beyond the volume of messages, participants also noted that they were often ill-suited to deal with the content and urgency of messages, particularly when receiving messages at inopportune times or under scenarios in which they did not have adequate support and resources to respond. Additionally, they reported that clinic staff were inadequately prepared to reduce the message burden on physicians ( Table 2 ). Even if weekly time was allotted for messaging (In Basket) work, the volume and urgency of the messages precluded physicians from handling them only during those times, with one physician suggesting, “I would like to have time built into our schedules for the In Basket work on a daily basis … We need to have an hour built into any day just to do that In Basket.”

When documenting visits, some participants expressed that they preferred self-written notes because they had concerns that relying on an assistant or scribe would slow workflows due to misunderstandings and required training time. In addition, scribes were not always available for physicians who preferred them. Several physicians also expressed that helping complex patients (i.e., those requiring more time in the EHR to accurately capture the presenting concern) provided high professional fulfillment.

Other physicians highlighted a potential discrepancy between EHR use measures and their experience. Several physicians stated that they prefer (when appointment time allows) to talk through their thought process with patients while they go through the process of writing notes or placing orders, resulting in longer measured EHR active use time, but also higher professional fulfillment and quality patient care.

Several participants offered potential solutions to the challenges they noted, including qualified staff and dedicated time for messaging (In Basket) management: “… they're doing things like they're hiring an RN, which is wonderful because RNs are not cheap, to try to offload us in the inbox. That's helpful. Every little bit is helpful.” and “I would like to have time built into our schedules for the In Basket work on a daily basis. We need that.”

#### EHR-Related Burdens

Several physicians expressed mixed feelings when describing the tension between the benefits of the EHR and the additional burdens it provides. Physicians appreciated the accessibility and organization of the EHR compared with previous systems, but strongly disliked how much time certain functions required.

In particular, physicians commented on the ability of the EHR to expand access to patients, particularly during the COVID-19 pandemic, with many expressing that the way in which it was implemented increased physician burden ( Table 2 ). Implementation without adequate support appeared to add to EHR-related burdens for primary care physicians and managers.

#### Turnover and Insufficient Staffing

Interview participants expressed that although they continue to enjoy caring for individual patients, they were overwhelmed by the number of new patients due to physician turnover particularly when the new patients were complex. One physician reflected on the increase in patients and messages since the COVID pandemic as a tension between patients and systems ( Table 2 ). Another noted that excess patient load due to physician and staff turnover exacerbated the system aspect of managing patient load and demand ( Table 2 ).

Although none of the participants identified patients themselves as a cause of burnout, chaotic system pressures, and unrealistic expectations were identified as contributors ( Table 2 ). Several physicians expressed that healthcare provider team familiarity and clinician-patient continuity affected their ability to work efficiently and find professional fulfillment. One participant noted, “I think I see some providers really struggling with the excess burden, not only of inbox, but I think with the churn of the staff,” while another described it as, “… if my nurse is out on leave, then there are other nurses that are already assigned to pods that are covering us in addition. So, it is not the same triage or depth of triage that they can provide.”

Conversely, participants noted that increasing staff familiarity and experience could be particularly important for them to assist with message triaging and order preparation, “I feel like if we could somehow fix this issue of staff turnover, I think that would definitely help the workflow overall.”

#### Demands and Resources Alignment

Across the identified themes, several participants reflected that organizational demands do not align with their available resources and felt that their agency was limited, thereby contributing to poor professional fulfillment. Many expressed that the organization seemed to increase job demands by increasing patient appointments or enabling patient messaging to increase patient satisfaction, but this is often done without providing protected time or flexibility in these activities. Several physicians reminisced about this change stating: “we used to have flexibility in our schedule” or “we used to have an hour each half session for documenting, for calling, for doing refills, for doing all that. Now I'm lucky if I have my lunch hour.”

Although workload and time demands were frequently cited as burdensome, the burden was never attributed to the patients themselves. Even physicians who self-identified as experiencing burnout did not express symptoms of depersonalization directed toward their patients. In fact, several participants reflected that they continued to care for and enjoy their interactions with patients in the midst of experiencing excessive burden.

### Clinic Comparisons

Unblinded comparisons among clinics showed largely similar responses despite clinic-level burnout scores, with two key exceptions. Physicians who noted value misalignment as a source of distress represented the three clinics with high and increasing burnout scores; this was not observed with the other clinics. Physicians from the clinic with low stable burnout scores reported that their clinic provided a dedicated remote day of work to alleviate additional EHR burden, which was perceived as highly beneficial.

## Discussion

In this study of primary care physicians, we discovered congruence between participant qualitative responses and the EHR audit log parameters identified to predict burnout at the clinic level through a recently developed machine learning model. Participants reported a high degree of work burden related to messaging, challenges maximizing team-based care during times of heightened turnover, and overall high demands for physician time that limit flexibility. In addition, several participants described an imbalance between organizational demands and individual resources that were perceived to drive the movement toward increased burdens that physicians did not find professionally fulfilling. Strikingly, participant responses centered primarily around institutional factors, consistent with our findings that the quantitative model carried greater predictive power for identifying clinic-level burnout risk, and the conceptualization of burnout as a work-related phenomenon.

The majority of participants described the EHR message (In Basket) management as a particular source of distress, consistent with our prediction model finding that the number of automated/administrative messages per day was the feature with the highest importance for burnout symptom prediction. These findings align with a growing body of evidence on the amount of messaging burden and its associations with burnout. 21 26 46 47 48 Notably, respondents expressed concerns with the implementation of and support for In Basket work rather than the concept itself. In particular, complaints centered around (1) the volume of messages and the relative infrequency of important content, (2) the fact that messages continue to pile up even while seeing other patients or out of the clinic, (3) limited support from other staff who can meaningfully reduce the burden of the messages, and (4) misaligned expectations and use of messaging by patients, particularly during and following the COVID-19 pandemic. These findings highlight the importance of organizational leadership to carefully consider the balance between burdens and resources as a contributor to individual physician well-being and organizational health.

Team-based care has frequently been championed as a solution to many of the excess demands perceived by primary care physicians. 49 50 51 52 53 54 55 56 However, participants expressed several challenges to implementing effective team-based care, potentially explaining the seemingly counterintuitive finding of our prediction model in which note composition by team members is associated with higher burnout. In clinics where a team-based approach to patient care is employed, individual team members are not always educated or ideally suited to perform the tasks assigned to them. Regarding offloading excess messaging burden, several participants expressed that they (1) lacked dedicated support within their clinic, (2) had inconsistency in the individuals supporting them, or (3) had support teams that were misaligned with the tasks assigned to them (e.g., medical assistants triaging patient medical advice requests rather than registered nurses). These findings highlight the importance of maximizing team-based coordination and minimizing turnover as strategies to mitigate burnout.

However, participants expressed differing opinions on team-based documentation, with some stating that they preferred to write their own notes because they felt their notes were more efficient and accurate that way. Individual clinics may benefit from assessing their own teams to determine which of all possible interventions would be most high yield, including technology-based solutions such as ambient scribes. 57

Underlying the specific domains discussed by participants we noted two underlying aspects: control and change. Several physicians noted a lack of control as a particular burden, especially as related to scheduling clinic hours, balancing patient visits with other more clerical tasks, and determining how the quality of their care was measured—all of which were described as decisions imposed upon them by the system and are related to well-described threats to well-being and retention. 58 59 60 Related to this, many of the burdens were framed within the context of change from a prior state which was often viewed in a more favorable light. The most common example of this related to the increased volume of patient medical advice requests, although other expressed sources of undesired change included physician and staff turnover, and balance between in-person and telehealth visits. These findings highlight the importance of physician inclusion and organizational communication in practice modification decisions.

In aggregate, these findings provide valuable updates to the conceptual framework underlying the quantitative prediction model. 16 Several of the identified system and physician experience factors were reinforced and highlighted by study participants, including workflow, payment structure (particularly in relation to medical advice requests), teamwork culture, job demands, efficiency of practice, time allocation, administrative work, perceived inefficiency, and work-home conflict. Notable additions to the conceptual framework include a feedback loop wherein physician turnover exacerbates several system factors contributing to a chaotic environment and a feedback loop in which patient dissatisfaction (particularly in relation to scheduling availability) exacerbates physicians' professional dissatisfaction.

### Limitations

Study findings should be viewed in relation to the design. Although we sampled a variety of physicians and clinic managers from academic and community-based primary care clinics, experiences may differ for physicians who were invited but chose not to respond, whose clinic managers declined participation, or who work in other settings. Although the interviewers were not in hospital leadership roles, social desirability bias may have affected participants' responses. Interviews were conducted shortly after the intensive phase of the COVID-19 response, and ongoing practice transformation across the healthcare sector may shed additional insights about the evolving state of physician burden.

## Conclusion

Many EHR-related burdens quantifiable through EHR usage measures correspond with the experiences of primary care physicians, who reported burdens related to messaging, team-based care, inadequate time to complete work demands, and frequent changes. Developing and including additional related EHR usage measures into future quantitative machine learning approaches may improve predictive performance and inform directed interventions to reduce burnout risk.

## Clinical Relevance Statement

Primary care physicians report mixed feelings regarding the electronic health record (EHR) system, with notable benefits in addition to frequent burdens related to excessive messaging burden in comparison to time available, high note burden, ordering frustration, and high patient loads. Inefficiencies of teamwork, staff turnover, and personal-organizational values misalignment contributed to perceptions of frustration. EHR usage measures align with experiences and may quantify misalignment between demands and resources within primary care.

## Multiple-Choice Questions

1. Which of the following was a frequently cited burden by primary care physicians?
  - System latency
  - In Basket and documentation burden
  - Interoperability and communication with other health systems
  - Patient complexity
  **Correct Answer:** The correct answer is option b. In Basket and documentation burden. Primary care physicians frequently cited In Basket as a burden, with comments centering around the volume of messages received, the content of messages that they were not always equipped to deal with, and the continuous nature of new messages resulting in In Basket work continuing during nonclinic days and vacations. These findings are consistent with quantitative approaches, which found the volume of automated In Basket messages to be one of the strongest predictors of primary care physician burnout.
2. When implementing a new feature to expand access to care for patients, which of the following was identified by primary care physicians as an important but often overlooked consideration?
  - Advertising the new feature to all patients equitably.
  - Pilot testing the new feature with patients to improve usability.
  - Ensuring the institution provides physicians with adequate resources to support the new feature.
  - Integrating artificial intelligence capabilities in the new feature.
  **Correct Answer:** The correct answer is option c. Ensuring the institution provides physicians with adequate resources to support the new feature. Particularly for features such as patient portals that improve access to care, many physicians noted that although the improved access to care was helpful to the patients, the institution did not often provide adequate resources of protected time or staffing to respond to the increased demands. This increased demand without increased resources was perceived to promote an imbalance and increase the risk of burnout.
