# Supplementary material for: Qualitative Verification of Machine Learning-Based Burnout Predictors in Primary Care Physicians: An Exploratory Study
Source: Appl Clin Inform. 2025 Sep 5;16(4):1031–40. doi: 10.1055/a-2595-0415 (PMC12413275; doi:10.1055/a-2595-0415)
Supplement: Supplementary file 1 — Supplementary Material [file 10-1055-a-2595-0415_26959271.pdf]

Supplementary Material S1

A. Interview Guide

Burnout Predictor Model Interview Guide: Physicians and Clinic Managers

Introduction

Hi, my name is [insert name] and I work with the Evaluation Sciences Unit at Stanford University School of Medicine.

We are working to get insight into work-setting characteristics that influence physician well-being.

Today, we want to hear your perspective on how particular workplace characteristics, including how you use the EMR, influence your well-being to inform interventions that could be implemented to proactively prevent burnout.

I just want to mention that everything you share today will remain confidential. This means that we will not share your identity when sharing your insights with the team. Our conversations typically take 30 minutes, and you are free to skip questions or end the interview at any time. Please don't hesitate to speak up.

To be cognizant of your time, how long are you able to chat with me today? Do you have a hard stop I need to pay attention to?

Do you have any questions before we start?

Before we start, are you comfortable with me audio recording our confidential discussion? This would help me focus on you instead of taking notes.

–Ok, I am turning on the recorder now.

–Can you please say your name, the date, and that you consent to being recorded?

Appreciative Inquiry

Let's start off with the positive aspects of your role.

1. Broadly, what do you like about being a physician?

Patient Load

2. Walk me through your typical workday.
   I. What's the good, the bad, and the ugly?
3. Let's talk about your patient load.
   I. Do you typically see the same group of patients, or do you have several changing patients?
   II. What type of new patients do you see? Simple or complex?
      i. What makes them simple or complex?
      ii. How challenging is it to capture the complexity of patients in the EMR?
4. How much face-to-face time do you have with patients?
   I. How much of your appointment time is spent looking at the computer?
5. Tell me about your favorite or most fulfilling type of visit.
   I. How much of this visit do you get?
   II. How much would you want for this type of visit, or what would be a reasonable amount?

Notes, Messages, and Time

6. Broadly, how would you describe your experience with EMR?
   I. How much time do you think you spend on EMR per shift?
   II. How much would be reasonable?
7. What is your experience with EMR notes?
   I. What does your process look like?
      i. Who writes them? (dictated, transcribed, self-typed?)
      ii. When do you write them?
   II. What would be a reasonable time in terms of managing notes?
8. Let's talk about your experience with In Basket messages
   I. What's your process?
      i. When do you manage messages?
      ii. What works or doesn't work?
   II. What would be a reasonable In Basket volume?

 © 2025. The Author(s).

9. Let's talk more about when you complete notes and In Basket messaging:
    I. When is an appropriate or optimal time to handle notes and messaging?
    II. When is a less desirable time?
    III. What has been your experience completing notes or messages outside of your standard working hours?
        i. What contributes to the need to complete notes and messages outside of your working hours?
        ii. In a typical week, how often does this happen?
        iii. Are there specific times or patients where you find it more necessary?
10. How have your thoughts about notes and messaging changed over time?
    I. How about COVID/Fall 2020?
11. Is there something else you would change about your EMR workflow if you could?

Orders

12. Let's talk about orders.
    I. What is your experience with orders?
    II. What is your process?
    III. When does it take more time to complete orders? Are there certain types or situations that require more time?
        i. What is the impact of regulatory issues or concerns in your ordering process?
        ii. How about referral or prior authorization issues?
        iii. Is there something else that increases the time an order takes? ("Too many clicks," etc.)
13. What types of orders do you find more fulfilling? (For example, are top-of-license orders more fulfilling than simple orders?)
14. What do you feel would be a reasonable percentage of time to spend on orders?

Physician Distress

15. What has been your experience with burnout?
    I. What do you think contributes to burnout? (Slow and steady increase, specific experience…)
    II. How many of those contributing factors are captured by the EMR?
16. Have you discussed these issues with your colleagues?
    I. Have they expressed similar opinions/experiences? Any you didn't mention?
17. Does your clinic have programs or other resources focused on improving the clinical practice environment or reducing work burden?
    I. Are there any improvement interventions focused on EMR-related activities? (For example, reimbursement for EMR patient messaging)
    II. Ideally, what would these interventions look like?
    III. What have you found to be most effective?
    IV. What have you found to be the least effective?
    V. What factors limit the impact of these interventions?

Wrap Up

Thank you so much for speaking with us today. I just have a couple more questions to wrap up.

18. Is there something else you would like to add?
19. Are there any questions we should have asked?

 © 2025. The Author(s).

B. Codebook

Name	Sources
Burden	16
Administrative work burden	5
After-hours time	4
Compensation	2
Work burden	0
Burnout	20
Burnout interventions	17
Desired intervention	4
Effective intervention	4
Ineffective intervention	1
Change	17
Double-codes	0
Clinic-level	3
Individual-level	2
Network-level	5
Electronic health record	19
Efficiency (EHR)	3
Self-taught strategies (EHR)	0
Templates, shortcuts, presets (EHR)	3
Training (EHR)	2
Insurance-disability-regulatory workflows (EHR)	5
Messages (EHR)	6
Notes and charts (EHR)	5
Open notes-open results	2
Orders (EHR)	5
Results (EHR)	1
Physician experience	2
Administrative-management time	3
Career length-stage	2
Clinic tenure	1
Climate of silence	3
Control of practice	10
Family and gender issues	2
FTE	8
Interruption and workflow	2
Joy of practice	19
Patient panel	19
Patient deaths	0
Patient impact on physician	0
Inappropriate patient behavior	1
Patient expectations	2
Pet peeves and strong emotions	6
Physician health	3

(*Continued*)

(*Continued*)

Name	Sources
Scheduling	5
Self-care	0
Emotional needs	1
Physical needs	1
Telehealth	8
Top of license	3
Work-life balance	1
Quotes	14
Teamwork	20
Time	19
Work setting	13
Clinic Leadership	3
Clinic leadership change	1
Clinic resources	1
Organizational change	0
Performance metrics	1
Staffing	1
External assistance-staff	0
Internal assistance-staff	5
System connections and values alignment	4

 © 2025. The Author(s).
